# An interpretable ensemble structure with a non-iterative training algorithm to improve the predictive accuracy of healthcare data analysis

**DOI:** 10.1038/s41598-024-61776-y

**Published:** 2024-06-05

**Authors:** Ivan Izonin, Roman Tkachenko, Kyrylo Yemets, Myroslav Havryliuk

**Affiliations:** Lviv Polytechnic National University, Lviv, 79013 Ukraine

**Keywords:** Computational science, Computer science

## Abstract

The modern development of healthcare is characterized by a set of large volumes of tabular data for monitoring and diagnosing the patient's condition. In addition, modern methods of data engineering allow the synthesizing of a large number of features from an image or signals, which are presented in tabular form. The possibility of high-precision and high-speed processing of such large volumes of medical data requires the use of artificial intelligence tools. A linear machine learning model cannot accurately analyze such data, and traditional bagging, boosting, or stacking ensembles typically require significant computing power and time to implement. In this paper, the authors proposed a method for the analysis of large sets of medical data, based on a designed linear ensemble method with a non-iterative learning algorithm. The basic node of the new ensemble is an extended-input SGTM neural-like structure, which provides high-speed data processing at each level of the ensemble. Increasing prediction accuracy is ensured by dividing the large dataset into parts, the analysis of which is carried out in each node of the ensemble structure and taking into account the output signal from the previous level of the ensemble as an additional attribute on the next one. Such a design of a new ensemble structure provides both a significant increase in the prediction accuracy for large sets of medical data analysis and a significant reduction in the duration of the training procedure. Experimental studies on a large medical dataset, as well as a comparison with existing machine learning methods, confirmed the high efficiency of using the developed ensemble structure when solving the prediction task.

## Introduction

Healthcare in recent decades began to develop at a very rapid pace^1^. This was facilitated by the progress in the chemical and pharmacological spheres and the rapid growth of the information technologies area. In particular, the development of the Internet of medical devices and feature engineering allowed remote monitoring of the patient's condition^2^. It has brought many benefits both for hospitals (because there is no overcrowding) and for patients (because they can stay in their usual home conditions).

A large number of different devices collect various indicators of the patient's condition. Since most of such devices are portable, data is collected all the time, regardless of the activity type for the subject of observation. Accordingly, a massive amount of information is collected, and there is a need for effective processing to make correct and timely medical decisions^3^.

The modern development of computer science certainly outpaces all other fields in growth. Many models, methods, and tools for processing various data types, and large volumes, have been developed. Processing tabular data is not a new task. Most machine learning (ML) and artificial neural networks (ANN) can do this. However, the problem of combining the high accuracy of intellectual analysis with the high speed of operation of the selected tools in the case of large data processing comes to the fore.

In this case, linear machine learning methods are rarely used for analysis despite the high speed of their work. It is explained by the insufficient accuracy of their work and problems with generalization when analyzing vast amounts of data^4^. Non-linear machine learning methods partially eliminate this drawback. In addition, combining such methods in the ensemble provides higher accuracy than using every single ensemble model^5^. However, three of the four classical strategies for the ensembling machine learning methods: boosting, bagging, and stacking, among others, do not always provide sufficient prediction accuracy, especially in the case of extensive data analysis^6^. In addition, most of the existing methods included in the ensemble are iterative, requiring additional time spent on their implementation.

That is why **this paper aims** to develop a new method of approximating large datasets based on the cascading scheme of the linear non-iterative SGTM neural-like structure with non-linear input extension.

**The main contribution** of this paper can be summarized as follows:

1. we developed a new linear, non-iterative cascade ensemble based on SGTM neural-like structures with non-linear input extension for large data analysis;
2. we determined the optimal complexity model of the developed cascade structure and selected its hyperparameter, which ensured both an increase in the accuracy of its work and a reduction in its training time;
3. by comparison with other methods, we showed the efficiency of the implementation of the developed cascade structure both: in the accuracy of the large data analysis and in the duration of the procedure of its training.

**The practical value** of the proposed cascade structure is as follows:

- it provides a significant increase in the accuracy of large data approximation as a result of the combined use of non-linear expansion of inputs at each new node of the cascade structure and the principles of response surface linearization;
- it ensures the formation of the approximate response surface by polynomials of high degrees (implicitly) without significantly complicating the training procedure;
- it demonstrates a high speed of operation due to the use of a linear SGTM neural-like structure with a non-iterative training algorithm;
- it allows for a transition from the neural network's form to a direct polynomial representation of the proposed cascade structure (interpretability).

This paper has the following structure. The overview and analysis of the existing works are presented in section “State-of-the-Arts”. Section “A non-iterative SGTM-based cascade structure with nonlinear input extension” describes the architectures and training algorithms of the SGTM neural-like structures in unsupervised and supervised modes; the peculiarities of the Kolmogorov-Gabor polynomial; and the detailed description of the designed non-iterative SGTM-based cascade structure with non-linear input extension. Dataset descriptions, optimal parameters selection procedures, and obtained results are presented in Sect. “Modeling and Results”. Section “Comparison and Discussion” contains the results of the comparison with existing methods, discussion, and future research perspectives. Conclusions are presented in Sect. “Conclusions”.

## State-of-the-Arts

Among the four existing ensembling strategies of machine learning algorithms or ANNs, cascading is the least represented class of methods. Despite this fact, it provides the highest prediction or classification accuracy when solving various applied tasks^7^.

The main idea of this approach is to build a multistage system, where the output of the previous level of the constructed structure is used by its next level. The principle of response surface linearization, which is the basis of this step, ensures an increase in the prediction or classification accuracy during the solution of a specific task.

In the scientific literature, various approaches to the implementation of cascading are considered, which can be reduced to three main groups:

- cascading inside the topology of a particular ANN:

- composition of two-step cascading methods;

- composition of branched multistage systems.

The first approach is presented in^8–10^. The authors from^8^ developed a cascade-forward neural network topology for solving the forecasting task. In this case, the signals fed to the ANN input are also fed to the hidden layer of the developed ANN topology to form the outcome^10^. The advantage of such a step is that the proposed approach considers non-linear connections between the inputs and output and the linear component^11^. A similar method was applied in^9^. The non-linear input extension of a non-iterative ANN is based on *rbf* functions. Due to this, the accuracy of its work increases significantly^12^. In addition, the authors of this method also use the initial inputs and outputs of the rbf-layer to form the output signal of the ANN. It ensures consideration of both linear and non-linear relationships between inputs and output, which significantly increases the extrapolative properties of the developed ANN. However, this approach does not efficiently analyze large datasets^13^.

The second group of cascading methods is covered by^14,15^. The composition of the methods of this class consists of two serially connected artificial intelligence tools. They can be heterogeneous as in^15,16^, or homogeneous as in^15^. In particular, in^15,16^, the detection tasks in various application areas are considered. Authors from^15^ applied serially connected Random Forest and MLP to increase the accuracy of solving the DDoS attack detection task. Another, the more classical approach is proposed in^14^. The authors developed a scheme of two serially connected ANNs, particularly General Regression Neutral Networks. The main difference in the approach, in this case, is that the first level of the cascade is intended for predicting the target attribute, and the second is for predicting the error of the first one. The final procedure of summing up the results of the work of both members of the cascade structure ensures an increase in prediction accuracy. However, the main disadvantage of the cascade^14^ is that it is designed to analyze small data. In the case of large data analysis, GRNN becomes very large and slow, which imposes a number of limitations on its practical use.

The third group of cascading methods is presented in^17,18^. In^17^, the main advantages of building branched multistage systems are outlined. In this case, the ensemble is made constructively. Each of its subsequent elements takes into account the results of the work of the previous one. That is, the ensemble's composition involves adding a new node in the form of a new ANN or ML algorithm in case of unsatisfactory accuracy of the work of the previous level. In the case of an ensemble composition from^17^, it is necessary to consider all outputs from all its previous stages to form the result of the ensemble's work. Similar schemes were developed in^19–21^, where different types of ANNs are used as cascade nodes to solve various tasks. Taking into account the results of all previous levels of the cascade structure at its last node is quite expensive, and this drawback is eliminated in^18^. In particular, the authors of^18^ developed a cascade structure for processing biomedical datasets. It is based on the use of serially connected RBF-SVRs. This non-linear artificial intelligence tool ensures high work speed, and its use in a cascade structure increases prediction accuracy^22^. The composition of the ensemble in this case involves the serial connection of all SVRs with RBF-input extension, where the output of the previous member of the ensemble is taken into account only at the next node. In this case, the final result of the ensemble's work is provided by its last node, which reaches the required level of accuracy. Despite this fact, this method is focused on medium-sized datasets analysis, and the processing of extensive data requires the development of more accurate and fast methods for their research.

## A non-iterative SGTM-based cascade structure with nonlinear input extension

### A non-iterative SGTM-based neural-like structures

Neural-like structures based on the Successive Geometric Transformations Model (SGTM neural-like structure)^23^ regardless of the specific formulation of the task, are intended for approximating an arbitrary response surface represented by the available points-vectors of the training sample by means of non-iterative learning. Variants of direct approximation of non-linear surfaces by SGTM neural-like structures with non-linear synapses and non-linear activation functions of neural elements are possible. However, the most effective approach was based on the preliminary expansion of the primary inputs with some non-linear functions (members of the Kolmogorov-Gabor polynomial^23^, radial basis functions^9^, etc.) and the subsequent application of linear SGTM neural-like structure, which forms a hyperplane from the arguments (extended primary inputs)^9,23–25^.

Non-iterative approximation algorithms based on the Least Squares Method^26^ are known, which are inefficient for cases of almost degenerate problems. The use of SVD methods^27^ is associated with a significant increase in time delays and certain limitations regarding the characteristics of the data being processed. That is why we will consider the features of the Successive Geometric Transformations Model^24^, which has an apparent geometric interpretation and is the basis for constructing SGTM neural-like structures in various application modes.

Let us consider the Successive Geometric Transformations Model and the basics of its algorithm in the unsupervised mode.

Let the training data sample be given by the set of *N* vectors of realizations (observations) $\overline{x}^{(1)}$. An arbitrary component of the sample vector is $x_{i,j}^{(1)}$ for all $i = \overline{1,N}$, $j = \overline{1,n}$, where $i$ is a number of the vector and $j$ is a number of the component of the vector. Step-by-step transformations perform the training process in the *n*-dimensional space of realizations (*n* is the number of vector components). Because of performing a sequence of geometric transformations, an intermediate coordinate system of the Principal Components is formed. Its coordinate directions coincide with the longest axes of the scattering ellipsoid given by the realization points of the training sample. Therefore, the basis for the training procedure is a training sample (matrix of rows-vectors) of data, where each row corresponds to the point of the ellipsoid. For the implementation of the training process, it is needed to perform the following transformations successively^23,24^:

(1) We set the initial value of the transformation step number S = 1.

(2) We calculate the norm of each vector of realizations $\left\| {\overline{x}^{(S)} } \right\|$. From the number of rows of the matrix, we choose the base row $x_{b}^{(S)}$, the norm of which is the largest. The base row ensures the coincidence of the coordinate direction of the intermediate coordinate system with the longest axis of the scattering ellipsoid.

(3) We calculate the coefficient for each sample vector $k^{(S)}$, which is close in magnitude to the first Principal Component^24^:

1 $$k^{(S)} = \frac{{\overline{x}^{(S)} \times \overline{x}_{b}^{(S)} }}{{\left\| {\overline{x}_{b}^{(S)} } \right\|}}.$$

In the numerator (1), we have the scalar product of the current vector by the base vector, and in the denominator, the norm of the base vector.

(4) After performing the increment S = S + 1, from the elements of each row vector of the training matrix, we subtract the corresponding elements of the base row, multiplied by the coefficient calculated by (1). As a result of such a transformation, the dimension of the space of realizations is reduced by one by projecting the points of realizations onto a normal plane (hyperplane) that passes through the origin of coordinates:

2 $$\overline{x}^{(S)} = \overline{x}^{(S - 1)} - k^{(S - 1)} \overline{x}_{b}^{(S - 1)} .$$

(5) We proceed to step 2 of the algorithm.

6) Transformations following (1) and (2) correspond to the Gram-Schmidt orthogonalization procedure, and then after performing the step S = n, if the columns of the matrix of realizations are not linearly dependent, we will get a zero matrix:

3 $$\overline{x}^{(S + 1)} \equiv \left( 0 \right).$$

7) As a result of the transformations that make up the training procedure, we get a set of vectors $\overline{x}_{b}^{(S)}$.

8) In the application mode, according to formula (1), the values $k^{(S)}$ and sought components of vectors are calculated by inverse transformations:

4 $$\overline{x}^{(S)} = \overline{x}^{(S + 1)} + k^{(S)} \overline{x}_{b}^{(S)} ,\,{\text{where}}\,\,\overline{x}^{(S\max + 1)} \equiv (0).$$

**Figure 1:**
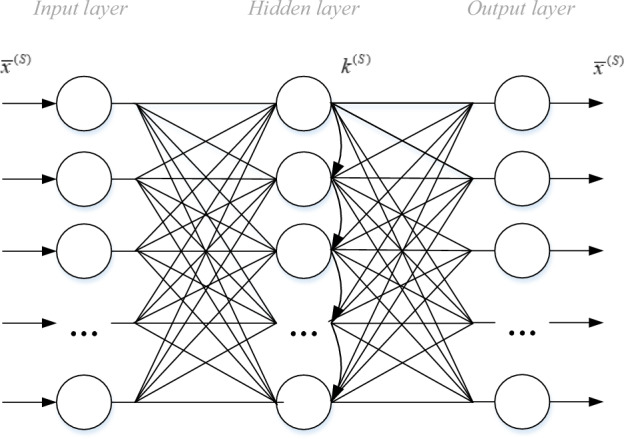
Topology of the SGTM neural-like structure in unsupervised mode.

The graph of the functioning algorithm of this model is presented in the form of a neural-like structure (Fig. 1).

A supervised mode of the SGTM neural-like structure^24^ is special in that we know the output components only for the vectors of the training sample. Therefore, to obtain the exact values of the coefficient $k^{(S)}$ it is necessary to perform a sequence of transformations only for the training vectors. In this case, it is enough to calculate the value of the coefficient $k^{(S)}$ based on (1) and present the sought coefficient as an approximation of dependence (5), which is given in the tabular form:

5 $$\tilde{k}^{(S)} = f(k^{(S)} )$$

**Figure 2:**
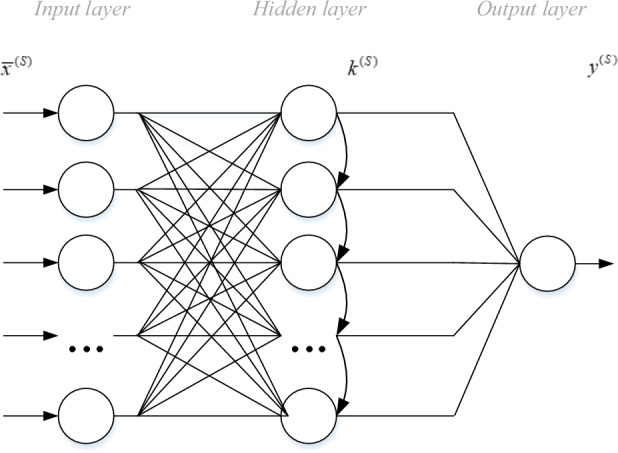
Topology of the SGTM neural-like structure in supervised mode.

The topology of the SGTM neural-like structure of this type is presented in Fig. 2. In this case, we can use linear or non-linear components in the hidden layer, the function of which is the approximation of dependence (5). For the case of the linear version of the SGTM neural-like structure, the type of activation function of the elements has the form:

6 $$\tilde{k}^{(S)} \approx \alpha^{(S)} k^{(S)}$$

where the coefficient $\alpha^{(S)}$ is calculated based on the least squares method.

### Kolmogorov–Gabor polynomial

The Kolmogorov-Gabor polynomial is a discrete analog of the Voltaire series^23,28^. It is widely used to approximate complex multiparameter dependencies^29,30^. In particular, it is used as a support function^31^ in the world-famous family of inductive methods for the self-organization of models in complex non-linear systems (the method of group consideration of arguments)^28–30^. This polynomial can be represented in the following form:

7 $$Y(x_{1} ,...,x_{n} ) = \theta_{i} + \sum\limits_{i = 1}^{n} {\theta_{i} x_{i} } + \sum\limits_{i = 1}^{n} {\sum\limits_{j = i}^{n} {\theta_{i,j} x_{i} x_{j} + \sum\limits_{i = 1}^{n} {\sum\limits_{j = i}^{n} {\sum\limits_{l = j}^{n} {\theta_{i,j,l} x_{i} x_{j} x_{l} } } } + ...} } + \sum\limits_{i = 1}^{n} {\sum\limits_{j = i}^{n} {\sum\limits_{l = j}^{n} {...\sum\limits_{z = k - 1}^{n} {\theta_{i,j,l,\,...,\,z\,} x_{i} x_{j} x_{l} \,...\,x_{z} } } } } ,$$

where $x_{i} ,\,\,\,\,x_{i} x_{j} ,\,\,\,x_{i} x_{j} x_{l} ,\,\,\,....\,\,\,,\,\,\,....\,\,,\,x_{i} x_{j} x_{l} \,....\,x_{z}$ are members of the Kolmogorov-Gabor polynomial; *n* is a feature number; $\theta$ are the polynomial coefficients; *k* is the Kolmogorov-Gabor polynomial degree $k = 1,\,\,2,\,\,3,\,\, \ldots$.

The authors in^23,28^ show the high efficiency of using the combination of the Kolmogorov-Gabor polynomial as a high-precision approximation tool for multiparameter non-linear dependencies and the SGTM neural-like structure as a high-speed tool for finding the coefficients of this polynomial. In^25^, the method of identifying the coefficients of the second-degree polynomial is developed by applying the diagonal matrix of test signals to the pre-trained SGTM neural-like structure. This approach provides a transition from a neural network form of model implementation to a polynomial one, which is faster, more trustworthy, and understandable to the user. In addition, using the Kolmogorov-Gabor polynomial of second degree to expand the inputs provided an optimal combination of accuracy and generalization. At the same time, it is the second degree provides the total number of inputs (N) for the SGTM neural-like structure acceptable for practical implementation^23,28^:

8 $$N = n + \frac{n(n + 1)}{2},$$

However, approximating significantly non-linear response surfaces requires using a higher degree of polynomials. The well-known Stone-Weierstrass theorem confirms this. In the case of expanding the inputs of the SGTM neural-like structure by members of this polynomial even of the third degree, there is an almost unrealistic increase in the number of inputs of the neural network. As a result, a deterioration of the generalization properties of the model. That is why there is a need, on the one hand—to ensure the accuracy and generalization of the model, which will use large degrees of the Kolmogorov-Gabor polynomial. On the other hand—to maintain as little growth as possible in the number of input variables of the task and, as a result, to reduce the duration of the training procedure of the SGTM neural-like structure.

### Proposed cascade structure

In this paper, we propose a new non-iterative SGTM-based cascade structure with a non-linear input extension to solve the above problem. Let us consider the method based on applying a cascade of serially connected SGTM neural-like structures with non-linear input extension (Fig. 1). It provides both a neural network form and a polynomial interpretation of the approximation model, equivalent to a high degree Kolmogorov-Gabor polynomial.

To implement the training procedures of the proposed method, we have to follow the following steps. Before executing the training algorithm for the developed non-iterative SGTM-based cascade structure, it is imperative to conduct data cleaning procedures (missing data recovery, removal anomalies, and outliers). To achieve heightened prediction accuracy, it is advisable to undertake feature selection and feature engineering procedures.

*Training mode*

Step 1. First of all, the training dataset should be partitioned into parts that will determine the number of levels of the non-iterative SGTM-based cascade structure. Each of them will be processed by its neural-like structure in the developed cascade structure. To simplify the description of the functioning of the designed structure, we will limit ourselves to the variant of the approximation of the two-dimensional dependence given by the training sample of the vectors $x_{1,i} ,x_{2,i} \to y_{i}$.

Step 2. At the second step of the training procedure, the inputs of the first SGTM neural-like structure of the cascade are expanded based on the first subsample using the quadratic Kolmogorov-Gabor polynomial. As a result, we will get an extended set of attributes for each data vector, based on which we train the first SGTM neural-like structure of the cascade:

9 $$x_{1,i} ,x_{2,i} ,x_{1,i}^{2} ,x_{2,i}^{2} ,x_{1,i} x_{2,i} \to y_{i} .$$

Thus, the first level of the developed cascade structure (*SGTM-1*) is trained. It should be noted that the inputs of the quadratic expansion for arbitrary dimensionality of the signals are formed similarly to (8) based on the use of (7).

Step 3. We operate with the second subsample in the third step (second level of the cascade). To do this, we use (8) and apply the extended dataset to the trained *SGTM-1*. At its output, we receive a signal $y_{1,i}$, which we add to this subsample as an additional attribute. Next, we implement the training of the second level of the developed cascade structure. In this case, after performing a quadratic expansion, we will apply form (9) vectors to the inputs of the second SGTM neural-like structure (SGTM-2) of the sequential graph.

10 $$x_{1,i} ,x_{2,i} ,x_{1,i}^{2} ,x_{2,i}^{2} ,x_{1,i} x_{2,i} ,y1_{i} ,y_{1,i}^{2} ,x_{1,i} y_{1,i} ,x_{2,i} y_{1,i} \to y_{i} .$$

We train *SGTM2* and proceed to the third step.

Step 4. We operate on the third subsample at the developed cascade structure's fourth training step. To do this, we use (8) and apply the extended dataset to the pre-trained *SGTM-1.* Next, we use (9) and apply the extended dataset to the trained *SGTM-2*. We get the predicted signal $y_{2,i}$, which we add to the third sample as an additional attribute. We perform a quadratic expansion of such a set and train the third SGTM neural-like structure (*SGTM-3*) of the sequential graph of the proposed structure.

Step 5. All subsequent steps (if necessary) are performed by analogy, starting from the first one. The last step (cascade level) will form the target value.

**Figure 3:**
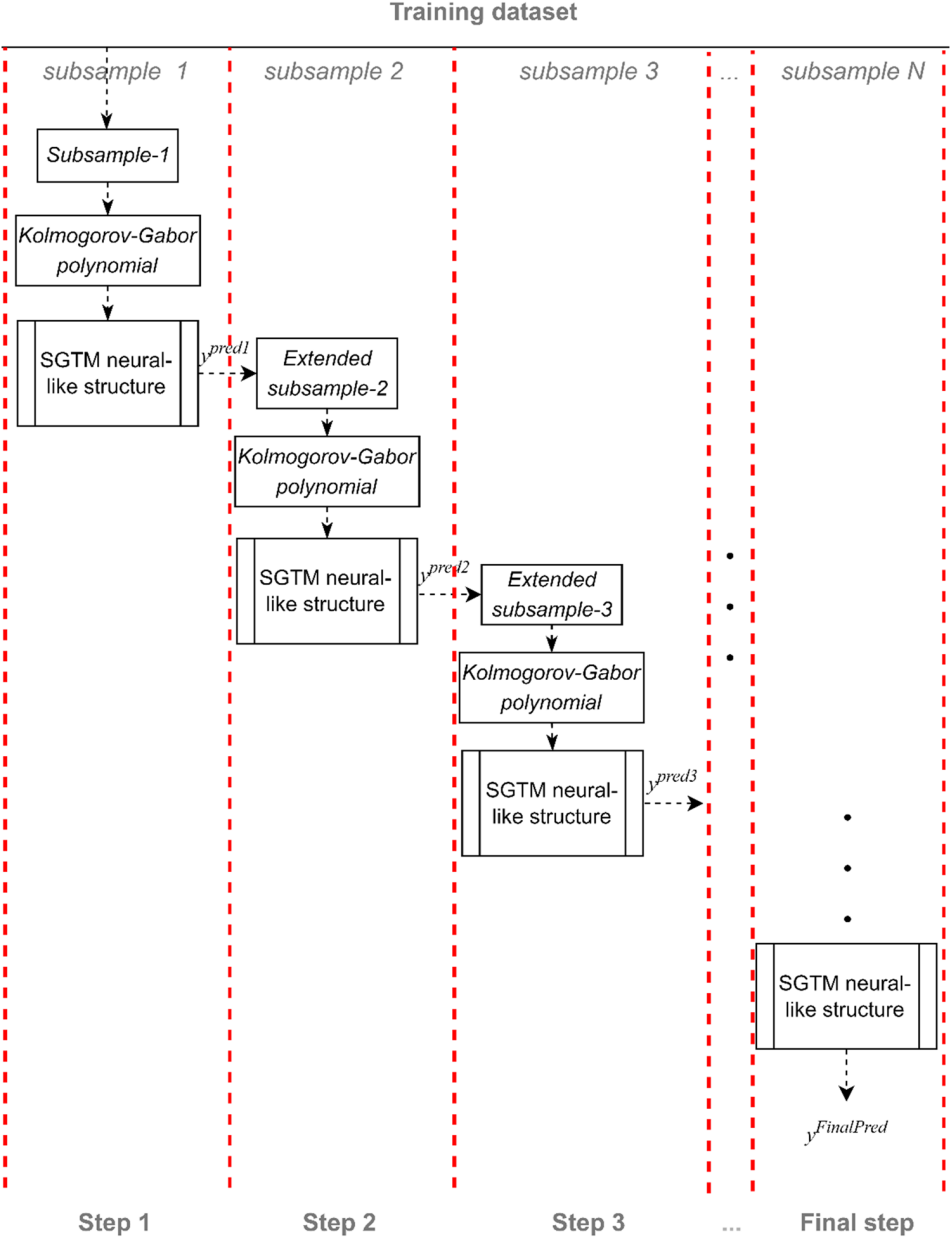
Flowchart of the proposed non-iterative SGTM-based cascade structure.

The detailed flowchart of the designed non-iterative SGTM-based cascade structure is shown in Fig. 3.

*Application mode*

The application mode of the proposed non-iterative SGTM-based cascade structure is similar to the training mode. For its implementation (based on a test sample or some specific vector), it is necessary to perform all the procedures described for the last step (cascade level) of the training mode of the proposed non-iterative SGTM-based cascade structure. That is to say, all components of the input vector with the unknown value of the desired attribute are augmented by a quadratic Kolmogorov-Gabor polynomial. Subsequently, the pre-trained *SGTM-1* model is applied to the extended vector in the initial stage of the cascade structure. The obtained predicted value is appended as an additional input attribute, and the test sample vector expanded in this manner is forwarded to the subsequent level of the cascade. Herein, once again, all components of the new vector undergo augmentation by the quadratic Kolmogorov-Gabor polynomial, and the pre-trained *SGTM-2* model, constituting the second-level cascade construction, is employed. These operations are iteratively repeated until reaching the final level of the cascade structure, culminating in the derivation of the ultimate predictive value for the desired output. It will be a neural network version of the proposed approach.

An essential advantage of the proposed approach is the possibility of transition from a neural network form to a direct polynomial representation of the proposed non-iterative SGTM-based cascade structure. This is possible because the signals at the output of the pre-trained *SGTM-1*, taking into account the expansions, can be described by an equivalent polynomial in the form:

11 $$y_{1,i} = a_{1,0} + a_{1,1} x_{1,i} + a_{1,2} x_{2,i} + a_{i,3} x_{1,i}^{2} + a_{1,4} x_{2,i}^{2} + a_{1,5} x_{1,i} x_{2,i}$$

Accordingly, the signals at the output of the pre-trained *SGTM-2* are described by a polynomial of the form:

12 $$\begin{gathered} y_{2,i} = a_{2,0} + a_{2,1} x_{1,i} + a_{2,2} x_{2,i} + a_{2,3} x_{1,i}^{2} + a_{2,4} x_{2,i}^{2} + \hfill \\ + a_{2,5} x_{1,i} x_{2,i} + a_{2,6} y_{1,i} + a_{2,7} y_{1,i}^{2} + a_{2,8} x_{1,i} y_{1,i} + a_{2,9} x_{2,i} y_{1,i} \hfill \\ \end{gathered}$$

If we substitute the value $y_{1,i}$ from expression (10) into expression (11), we will get a polynomial of the 4th degree as a result. Accordingly, substituting $y_{2,i}$ into the expression corresponding to the following *SGTM-N* of the developed cascade structure, we obtain a polynomial of the 8th degree, etc.

The coefficients from (10), (11), and others can be identified by applying the method of the diagonal matrix of test signals to the pre-trained SGTM neural-like structure, which is described in^25^. It will ensure the possibility of transition from the neural network version of the model application to the polynomial one.

In addition, the step-by-step increase in the cascade levels, and therefore the number of SGTM neural-like structures forming a consecutive graph, ensures the formation of the approximation surface by the resulting polynomial of high degrees without significantly complicating the training process.

However, if the number of input attributes is large, the construction of the resulting polynomial is inappropriate due to the too cumbersome writing formula. The neural network variant of the designed non-iterative SGTM-based cascade structure turns out to be more suitable for practical application.

## Modeling and results

### Dataset descriptions

Modeling of the proposed non-iterative SGTM-based cascade structure took place using a real-world set of large data. It is placed in an open repository^32^.

This large dataset was obtained from the electrocardiographic signals of various patients in different stressful conditions. It contains 18 independent attributes that affect a person's heart rate. The dependent attribute is the heart rate of individuals. The main statistics of the chosen dataset are presented in^18^.

In total, the dataset contains 369,289 observations. The task is to predict a person's heart rate based on the provided biomedical indicators. The dataset was divided into a ratio of 70% to 30% to form training and test samples.

### Modeling

The modeling of the developed structure took place using proprietary software developed in the Python language. We use the PC with the following characteristics: CPU-M1 Pro, 16 gb RAM.

Since the authors of the dataset previously cleaned it of omissions and outliers, pre-processing involved only data normalization. We use *MaxAbsScaler*() to normalize the data at each level of the cascading structure.

In addition, the second degree of the Kolmogorov-Gabor polynomial is used for modeling. It is explained by the fact that higher degrees of this polynomial significantly increase the number of attributes. It can provoke overfitting and problems with the generalization properties of the model^28^. In addition, higher degrees of this polynomial significantly increase the training time of the model^23^.

The effectiveness of the developed cascade structure, like any other machine learning method, depends on the optimal parameters of its operation. Among the operating parameters of the developed cascade structure, the following should be highlighted:

- the optimal number of the cascade structure's levels;
- hyperparameter of the SGTM-based neural-like structure.

A number of experimental studies were conducted to select them, the results of which are presented below.

(1) Optimal cascade level selection

The developed cascade structure is based on the principle of response surface linearization. It ensures a significant reduction in the operating errors of the proposed model. In this mode, the accuracy of the developed cascade structure will increase as the number of cascade levels increases. However, there may be a problem with the application mode. The generalization properties of the cascade structure can significantly deteriorate with a significant increase in the cascade levels. That is why this experiment aims to determine the optimal number of cascade levels, that is, to select the so-called "optimal complexity model" (according to the theory of Academician O. Ivakhnenko^31^). On the one hand, the cascade structure should ensure the slightest error of operation, and on the other hand—high generalization properties.

We conducted experimental studies on constructing a non-iterative SGTM-based cascade structure of different levels (from 1 to 4). The results obtained based on four various errors in the training and application modes, as well as the duration of the implementation of the training procedures, are shown in Table 1.

**Table 1:** Operation errors of the proposed structure when using different numbers of cascade levels.

Cascade levels	Maximum residual error	Mean absolute error	Mean squared error	Median absolute error	Training time, seconds
Training mode					
**1**	1.808	0.143	0.046	0.096	62.1
**2**	1.361	0.056	0.008	0.036	87.5
**3**	0.948	0.052	0.007	0.034	100.0
**4**	0.969	0.050	0.006	0.033	114.8
Test mode					
**1**	2.163	0.146	0.049	0.097	–
**2**	1.514	0.056	0.008	0.036	–
**3**	1.400	0.053	0.007	0.034	–
**4**	1073.098	0.071	15.511	0.034	–

**Figure 4:**
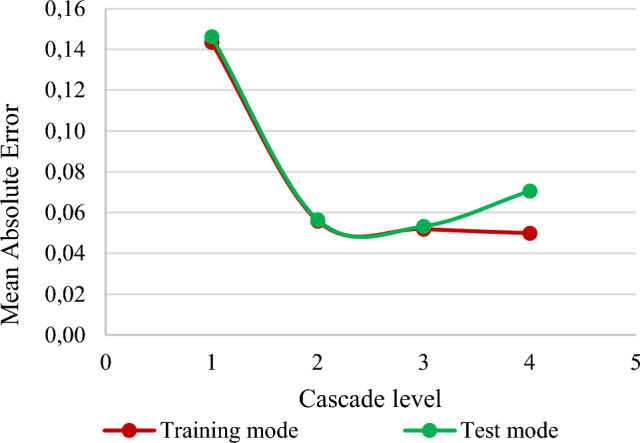
The optimal complexity model for the developed cascade structure.

As can be seen from Table 1, the theoretical analysis presented above fully coincides with the obtained results. That is, the values of all four operating errors of the cascade structure show a decrease with each new level of the cascade (training mode). However, this process takes place up to a certain point, when the model begins to lose its generalization properties. It is clearly visible in the application (test) mode performance indicators. In particular, as seen in Table 1, the error of the application mode of the cascade structure decreases to the third level of the cascade. Starting from the fourth level, the values of all performance indicators increase significantly. For ease of analysis, this process using the MAE of both operating modes for different number of cascades is shown in Fig. 4.

Figure 4 clearly shows that the optimal complexity model for the designed non-iterative SGTM-based cascade structure is obtained at three cascade levels. The such structure provided (implicitly) the eighth-degree approximation of the Kolmogorov-Gabor polynomial.

In addition to the high prediction accuracy, such a small number of cascade levels also ensures the need to process a significantly smaller number of features and, as a result, a reduction in the duration of the training procedure in comparison with the use of a larger number of cascade structure's levels.

(2) SGTM neural-like structure hyperparameter selection

The developed cascade structure is based on the non-iterative SGTM neural-like structure. In terms of ensemble learning, this is a weak predictor. As mentioned in the previous section, the SGTM neural-like structure topology is formed based on the input data. In this case, the number of neurons in the input and hidden layers is equal.

In addition to the response surface linearization, which significantly increases the accuracy of the cascade structure, the basis of the proposed structure is also the use of the Kolmogorov-Gabor polynomial. This tool for non-linear input extension provides high approximation properties of the model but significantly expands the input space of the task. Accordingly, the number of neurons of the input and hidden layers of the non-iterative SGTM neural-like structure increases significantly.

A feature of the non-iterative SGTM neural-like structure is that in the hidden layer, values ​​are formed that are very close to the principal components^24^. In addition, according to the training algorithm, they are immediately sorted in descending order^24^. The latter values ​​are minimal and can add noise components to the model. That is why the only parameter that can affect the accuracy of the work of the non-iterative SGTM neural-like structure, and accordingly, which should be adjusted, is the number of neurons of the hidden layer. A slight decrease in them can eliminate the influence of noise components, which will increase the accuracy of the model in general. However, the main advantage in our case will be a reduction in the duration of the training procedure.

**Figure 5:**
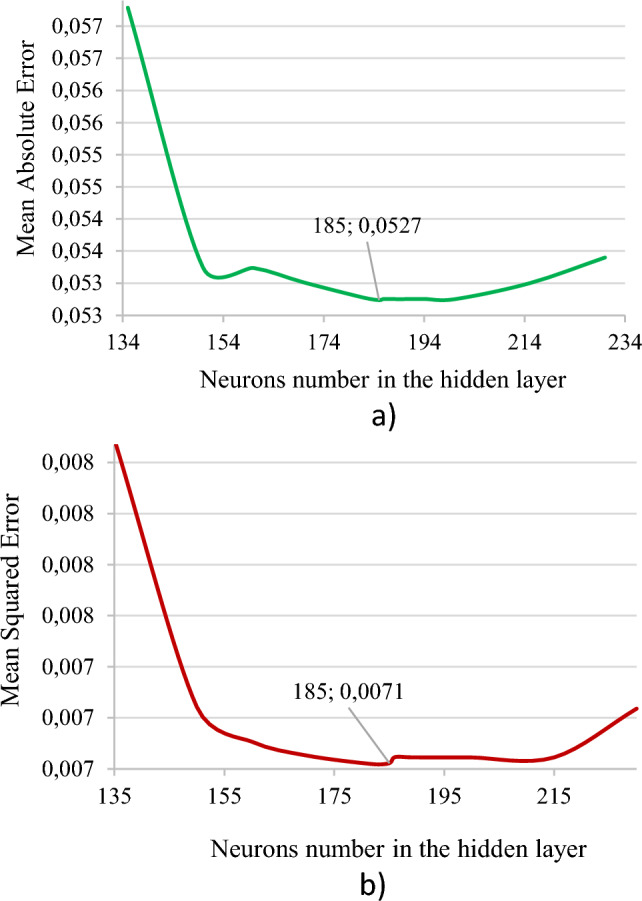
Errors values of the designed non-iterative SGTM-based cascade structure when changing the number of hidden layer neurons: (**a**) MAE, (**b**) MSE.

In the paper, experiments were conducted on selecting the optimal number of neurons of the hidden layer of the non-iterative SGTM neural-like structure when expanding the input data space by the Kolmogorov-Gabor polynomial of the second degree. The experiment was carried out using the brute-force method on the interval [50: 230] with a step of 1 while using a cascade structure of three levels. For better visualization of the results, Fig. 4 shows the error values ​​starting with 134 neurons in the hidden layer. A significantly smaller number of them demonstrates the unsatisfactory accuracy of the work and reduces the informativeness of Fig. 5.

As seen from both graphs in Fig. 5, values of the number of neurons of the hidden layer less than 155 significantly increase the errors of the non-iterative SGTM neural-like structure and are unacceptable. Values of this indicator greater than 217 slightly reduce the accuracy of this machine learning tool. The best accuracy was obtained using 185 neurons in the hidden layer of the SGTM neural-like structure. Therefore this value will be used in the future during the practical implementation of the developed cascade structure.

## Results

The results for the designed non-iterative SGTM-based cascade structure based on the optimal parameters of its work, using fivefold cross-validation are summarized in Table 2.

**Table 2:** Prediction results of the non-iterative SGTM-based cascade structure with its optimal parameters after fivefold cross-validation.

Errors	Training mode	Test mode	Optimal parameters
Maximum Residual Error	0.939 ± 0.01	1.312 ± 0.02	Cascade with 3 levels; 230 neurons in the input layer, 184 neurons in the hidden layer of the SGTM neural-like structure
Mean Absolute Error	0.049 ± 0.002	0.051 ± 0.002	
Mean Squared Error	0.006 ± 0.0007	0.007 ± 0.0005	
Median Absolute Error	0.0315 ± 0.006	0.0324 ± 0.008	
Training time, seconds	80,02	-	

The following conclusions can be drawn from the modeling and results summarized in Table 2:

- the designed non-iterative SGTM-based cascade structure due to the use of the Kolmogorov-Gabor polynomial and the principle of response surface linearization provides very high accuracy of approximation of large datasets;
- 5-folds cross-validation results show that the proposed approach is independent of the distribution of data into training and test samples, where, in particular, the deviation of the maximum error varies within 0.01;
- non-linear input extension of each level of the cascade by members of the Kolmogorov-Gabor polynomial of the second degree does not lead to the model overfitting. The values of all performance indicators in the training mode are lower than in the application mode;
- using the principle of response surface linearization, we obtained very high generalization properties of the developed cascade structure. The difference between the values of the corresponding errors in the training and application modes is minimal;
- determination of the optimal complexity model of the developed cascade structure ensured the construction of its optimal structure, which provides, on the one hand, the highest approximation accuracy and, on the other hand, the highest generalization properties;
- determination of the optimal number of neurons of the hidden layer in the cascade structure provided a slight increase in the accuracy of the entire cascade structure and a significant reduction in its training time. In particular, the training time without selecting this hyperparameter was 100 s (Table 1), and after reducing the number of neurons of the hidden layer – 80 s (Table 2).

## Comparison and discussion

The effectiveness of the designed non-iterative SGTM-based cascade structure was compared with several well-known and similar methods. We chose some linear methods because the developed cascade is also based on the linear machine learning method. They ensure high speed of training on large datasets. A number of non-linear methods were also chosen due to the high accuracy of their work. In addition, ensemble methods are taken as methods of the class to which the developed cascade structure belongs.

Among linear ones, we should highlight basic SGTM neural-like structures, Ridge-, Linear-, Bayesian Ridge-, ARD- Regressors, and SVR with linear kernel^33^. Among the non-linear ones are an extended-input SGTM neural-like structure, SVR with *rbf*- and *polynomial* kernels. Comparisons have also been made to the Gradient Boosting method, similar to a proposed cascade structure. In addition, for comparison, a similar cascade scheme is taken, which is also based on the principle of response surface linearization and non-linear expansion of the inputs^18^. However, in this case, the non-linear input extension takes place using *rbf*-functions, and the basic regression algorithm is SVR.

The performance results of all considered methods based on four errors (Maximum Residual Error—MRE, Mean Absolute Error—MAE, Mean Squared Error—MSE, Median Absolute Error—MedAE) with the optimal parameters of their operation are summarized in Table 3.

**Table 3:** Comparison with other techniques.

Method	Maximum residual error	Mean absolute error	Mean squared error	Median absolute error
Proposed cascade scheme	1.312	0.051	0.007	0.032
SVR(rbf)	14.454	0.063	0.041	0.048
SVM(rbf)-based cascade scheme	11.984	0.110	0.077	0.062
Extended-input SGTM neural-like structure	2.183	0.146	0.048	0.097
Gradient Boosting Regressor	6.412	0.343	0.264	0.227
SVR(linear)	10.516	1.065	3.007	0.607
SGTM neural-like structure	9.526	1.121	2.676	0.778
LinearRegression	9.520	1.121	2.676	0.779
ARD Regressor	9.539	1.121	2.676	0.779
Bayesian Ridge Regressor	9.542	1.121	2.677	0.780
Ridge Regressor	9.552	1.122	2.678	0.783
SVR(poly)	43.11	1.652	5.928	1.121

Let us consider the results of the comparison in more detail. The worst results in terms of Maximum Residual Error are shown by linear, non-linear, and cascaded SVR-based machine learning methods with different kernels. It is explained by several disadvantages of this machine learning method in the case of processing large datasets. Our cascade structure demonstrates more than 9 times better accuracy than the most similar method—SVR(*rbf*) cascade scheme.

The analysis of the other three error indicators from Table 3 shows that all linear methods are significantly inferior in accuracy to non-linear ones. It should be noted here that the linear SGTM neural-like structure^24^, which is the basis of the developed cascade, shows more than 10 times lower accuracy than the extended-input SGTM neural-like structure^28^. The peculiarity of the latter is that it processes a non-linearly extended dataset based on the Kolmogorov-Gabor polynomial of the second degree. It is another argument favouring this polynomial for non-linear input extension. The development cascade structure, due to the response surface linearization, demonstrates more than 3 times higher accuracy (based on various errors) than the extended-input SGTM neural-like structure^28^ that processes the entire dataset.

If we consider the most similar methods to the developed cascade structure, then SVR(*rbf*) cascade scheme shows 9 times, and the Gradient Boosting Regressor more than 32 times higher MSE error value compared to the developed method. It should be noted that the SVR(*rbf*) cascade scheme shows lower accuracy than the classical SVR(*rbf*) with optimally selected hyperparameter values.

Summarizing the obtained results for all studied methods, it can be stated that the combined use of the non-linear input extension by the Kolmogorov-Gabor polynomial of the second degree and the principles of response surface linearization provide the highest accuracy of the developed structure among those considered. However, an important indicator of the effectiveness of the ML methods application during the analysis of large datasets is the duration of their training procedure.

**Figure 6:**
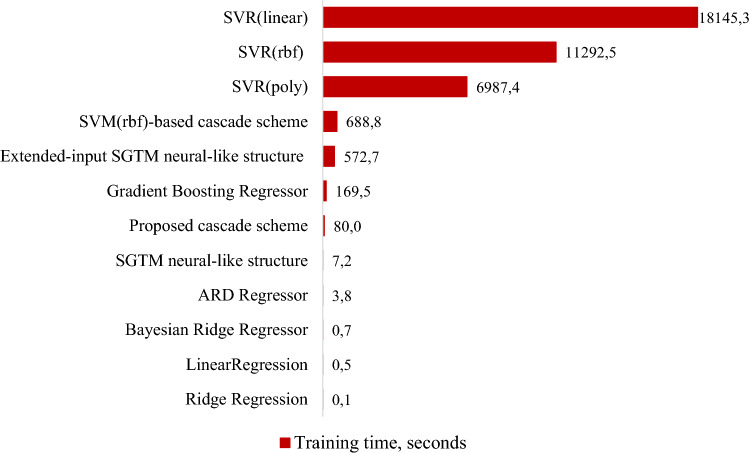
The duration (in seconds) of the training procedure for all studied methods.

In Fig. 6 summarizes the results of the training procedures duration of all considered methods.

As can be seen from Fig. 6, linear methods show the lowest running time, and SVR-based methods show the highest one. The developed cascade structure shows almost 8 times less training time than the most similar cascade—SVR(*rbf)* cascade scheme and more than 2 times less training time than the boosting ensemble. It should be noted here that it shows a significantly more accurate result of the approximation of large datasets. It is explained by the peculiarities of the SGTM neural-like structure's non-iterative training procedure, which is the basis of the developed cascade structure.

Despite this, it is necessary to consider the possibility of reducing the duration of the training procedure for the developed scheme. In particular, this can be achieved by utilizing the SGTM neural-like structure in the unsupervised mode of operation (Fig. 1) at each cascade level after expanding the input data space using a polynomial. This type of SGTM neural-like structure enables the transition to the principal component space, significantly reducing the input data space for each regressor in the cascade by discarding a large number of insignificant principal components. This approach has the potential to significantly decrease the training time of the entire cascade structure while maintaining high accuracy in its operation.

Furthermore, in the case of processing middle-sized medical datasets, where each regressor in the cascade receives considerably less useful information for the training procedure, there is a potential for enhancing prediction accuracy through cascade construction. In this scenario, the available dataset would be divided not into equal parts like in the proposed scheme, but into substantially larger data subsamples with repetitions, ensuring improved accuracy of each regressor's operation within the cascade and consequently, of the developed method as a whole.

Among the prospects for further research, it is worth considering the possibility of employing other linear machine learning techniques and artificial neural networks as weak predictors within the cascade structure. Such an approach would also demonstrate improved prediction accuracy across various medical domains; however, it would take into account the potential advantages of applying specific linear methods to address the stated medical task.

## Conclusions

In this paper, a new non-iterative SGTM-based cascade structure with non-linear input extension is developed for a fast and accurate approximation of large data. It is based on applying a cascade of serially connected SGTM neural-like structures with non-linear input extension. Members of the Kolmogorov-Gabor polynomial of the second degree realized the last one. The training algorithm of the developed structure is described in detail, and the flowchart of its realization is presented.

Modeling of the non-iterative SGTM-based cascade structure with non-linear input extension was carried out on a real-world set of large data collected by an electrocardiography tool for predicting the heart rates of humans. It can be noted the following from the obtained results:

- the designed non-iterative SGTM-based cascade structure due to the use of the Kolmogorov-Gabor polynomial and the principle of response surface linearization provides very high accuracy of approximation of large datasets. In particular, the test error of the proposed structure based on MSE is 5.94 times lower than the basic method and 9.54 times lower than the closest existing cascade method;
- non-linear input extension of each level of the cascade by members of the Kolmogorov-Gabor polynomial of the second degree does not lead to the model overfitting. The values of all performance indicators in the training mode are lower than in the test mode. In addition, the training time of the proposed structure is more than 7 times lower than the basic method and 8 times lower than the closest existing cascade method;
- the use of the principle of response surface linearization obtained very high generalization properties of the developed cascade structure. The difference between the values of the corresponding errors in training and test modes is minimal (from 0.001 for MAE to 0.3 for MRE);
- determination of the optimal complexity model of the developed cascade structure (3 levels of the cascade) ensured the construction of its optimal structure, which provides, on the one hand, the highest approximation accuracy and, on the other hand, the highest generalization properties;
- determination of the optimal number of neurons of the hidden layer in the cascade structure provided both a slight increase in the accuracy of the entire cascade structure and a significant reduction in its training time. In particular, the training time without selecting this hyperparameter was 100 s (Table 1), and after reducing the number of hidden layer neurons—80 s (Table 2).

An essential advantage of the proposed approach is the possibility of transition from a neural network form to a direct polynomial representation of the proposed non-iterative SGTM-based cascade structure. Therefore, the proposed approach can be classified as an interpretable AI solution^34^. However, if the number of input attributes is large, the construction of the resulting polynomial is inappropriate due to the too cumbersome writing formula. In this case, the neural network variant of the designed non-iterative SGTM-based cascade structure is more suitable for practical use.

The shortcomings of the proposed non-iterative SGTM-based cascade structure, which are ultimately characteristic of all ensemble methods from the cascading class, is the impossibility of implementing parallelization to speed up its work^14^. However, the use of the high-speed SGTM neural-like structure partially reduced the problems of the speed of the cascade structure due to the non-iterative nature of the training algorithm. However, among the prospects of further research, we will consider the PCA analogs, in particular, using^24^ to extract the principal components after the quadratic expansion of the inputs at each level of the cascade. This approach can significantly reduce the space of input data of the problem, which significantly reduces the duration of the training procedure of the entire cascade structure.

## Data Availability

The data used in this paper is in an open repository https://www.kaggle.com/datasets/vinayakshanawad/heart-rate-prediction-to-monitor-stress-level.

## References

[1] Chumachenko D, Piletskiy P, Sukhorukova M, Chumachenko T (2022). Predictive model of Lyme disease epidemic process using machine learning approach. Appl. Sci..

[2] Krak, I., Barmak, O., Manziuk, E. & Kulias, A. Data classification based on the features reduction and piecewise linear separation. in *Intelligent Computing and Optimization* (eds. Vasant, P., Zelinka, I. & Weber, G.-W.) vol. 1072 282–289 (Springer International Publishing, Cham, 2020).

[3] Berezsky, O., Pitsun, O., Liashchynskyi, P., Derysh, B. & Batryn, N. Computational intelligence in medicine. in *Lecture Notes in Data Engineering, Computational Intelligence, and Decision Making* (eds. Babichev, S. & Lytvynenko, V.) vol. 149 488–510 (Springer International Publishing, Cham, 2023).

[4] Mulesa O, Geche F, Nazarov V, Trombola M (2019). Development of models and algorithms for estimating the potential of personnel at health care institutions. EEJET.

[5] Geche, F., Mitsa, O., Mulesa, O. & Horvat, P. Synthesis of a two cascade neural network for time series forecasting. in *2022 IEEE 3rd International Conference on System Analysis & Intelligent Computing (SAIC)* 1–5 (IEEE, Kyiv, Ukraine, 2022). doi:10.1109/SAIC57818.2022.9922991.

[6] Ortega LA, Cabañas R, Masegosa AR (2021). Diversity and Generalization in Neural Network Ensembles..

[7] Paul, S. Ensemble Learning—Bagging, Boosting, Stacking and Cascading Classifiers in Machine Learning…. *Medium* https://medium.com/@saugata.paul1010/ensemble-learning-bagging-boosting-stacking-and-cascading-classifiers-in-machine-learning-9c66cb271674 (2019).

[8] Warsito B, Santoso R, Suparti, Yasin H (2018). Cascade forward neural network for time series prediction. J. Phys.: Conf. Ser..

[9] Tkachenko, R., Kutucu, H., Izonin, I., Doroshenko, A. & Tsymbal, Y. Non-iterative neural-like predictor for solar energy in Libya. in *ICTERI2018* (eds. Ermolayev, V. et al.) vol. 2105 35–45 (CEUR-WS.org, Kyiv, Ukraine, 2018).

[10] Banerjee A, Pohit G, Panigrahi B (2017). Vibration analysis and prediction natural frequencies of cracked timoshenko beam by two optimization techniques - Cascade ANN and ANFIS. Mater. Today: Proc..

[11] Abd-Elmaboud ME, Abdel-Gawad HA, El-Alfy KS, Ezzeldin MM (2021). Estimation of groundwater recharge using simulation-optimization model and cascade forward ANN at East Nile Delta aquifer, Egypt. J. Hydrol.: Region. Stud..

[12] Subbotin S (2020). Radial-basis function neural network synthesis on the basis of decision tree. Opt. Mem. Neural Netw..

[13] Islam, Md. F. & Oo, A. M. T. Modified cascade-correlation of ANN for short term prediction of wind speed. in *Power and Energy Systems* (ACTAPRESS, Phuket, Thailand, 2010). 10.2316/P.2010.701-038.

[14] Tkachenko, R., Izonin, I., Dronyuk, I., Logoyda, M. & Tkachenko, P. Recover missing sensor data with GRNN-based cascade scheme. *Int. J. Sensors Wireless Commun. Control* 1–10 (2020).

[15] Gholampour, I. & Nayebi, K. High performance telephony speech recognition via cascade HMM/ANN hybrid. in *ISSPA ’99. Proceedings of the Fifth International Symposium on Signal Processing and its Applications (IEEE Cat. No.99EX359)* vol. 2 645–648 (Queensland Univ. Technol, Brisbane, Qld., Australia, 1999).

[16] Pinto, T. & Sebastian, Y. Detecting DDoS attacks using a cascade of machine learning classifiers based on Random Forest and MLP-ANN. in *2021 IEEE Madras Section Conference (MASCON)* 1–6 (IEEE, Chennai, India, 2021). 10.1109/MASCON51689.2021.9563266.

[17] García-Pedrajas, N., Ortiz-Boyer, D., del Castillo-Gomariz, R. & Hervás-Martínez, C. Cascade ensembles. in *Computational Intelligence and Bioinspired Systems* 598–603 (Springer, Berlin, Heidelberg, 2005). 10.1007/11494669_73.

[18] Izonin, I. *et al.* Multistage SVR-RBF-based model for heart rate prediction of individuals. *AIMEE2022: The 6th International Conference of Artificial Intelligence, Medical Engineering, Education August 19 - August 21, 2022 , Wuhan, China* (in press).

[19] Bodyanskiy, Ye. V., Tyshchenko, O. K. & Boiko, O. O. An evolving cascade system based on neuro-fuzzy nodes. *Radio Electronics, Computer Science, Control*, (2016).

[20] Zaychenko, Y. P. & Hamidov, G. Cascade neo-fuzzy neural network in the forecasting problem at stock exchange. *SRIT* **0**, 92–102 (2017).

[21] Borenović, M., Nešković, A. & Budimir, D. Cascade-Connected ANN Structures for Indoor WLAN Positioning. in *Intelligent Data Engineering and Automated Learning - IDEAL 2009* (eds. Corchado, E. & Yin, H.) vol. 5788 392–399 (Springer Berlin Heidelberg, Berlin, Heidelberg, 2009).

[22] Dobrescu E, Nastac D-I, Pelinescu E (2014). Short-term financial forecasting using ANN adaptive predictors in cascade. Int. J. Process Manag. Benchmark..

[23] Tkachenko R, Izonin I, Vitynskyi P, Lotoshynska N, Pavlyuk O (2018). Development of the non-iterative supervised learning predictor based on the Ito decomposition and SGTM neural-like structure for managing medical insurance costs. Data.

[24] Tkachenko, R. & Izonin, I. Model and Principles for the Implementation of Neural-Like Structures Based on Geometric Data Transformations. in *Advances in Computer Science for Engineering and Education* (eds. Hu, Z., Petoukhov, S., Dychka, I. & He, M.) vol. 754 578–587 (Springer International Publishing, Cham, 2019).

[25] Izonin, I., Tkachenko, R., Kryvinska, N., Tkachenko, P. & Greguš ml., M. Multiple Linear Regression Based on Coefficients Identification Using Non-iterative SGTM Neural-like Structure. in *Advances in Computational Intelligence* (eds. Rojas, I., Joya, G. & Catala, A.) 467–479 (Springer International Publishing, Cham, 2019). 10.1007/978-3-030-20521-8_39.

[26] Dimitrov DK, Peixoto LL (2020). An efficient algorithm for the classical least squares approximation. SIAM J. Sci. Comput..

[27] Guan Y, Chu MT, Chu D (2018). SVD-based algorithms for the best Rank-1 approximation of a symmetric tensor. SIAM J. Matrix Anal. Appl..

[28] Vitynskyi, P., Tkachenko, R., Izonin, I. & Kutucu, H. Hybridization of the SGTM Neural-Like Structure Through Inputs Polynomial Extension. in *2018 IEEE Second International Conference on Data Stream Mining & Processing (DSMP)* 386–391 (IEEE, Lviv, Ukraine, 2018). doi:10.1109/DSMP.2018.8478456.

[29] Kalina J, Neoral A, Vidnerová P (2021). Effective automatic method selection for nonlinear regression modeling. Int. J. Neur. Syst..

[30] Щeлкaлин BH (2015). A systematic approach to the synthesis of forecasting mathematical models for interrelated non-stationary time series. EEJET.

[31] Ivakhnenko, A. G. Polynomial Theory of Complex Systems. *IEEE Transactions on Systems, Man, and Cybernetics* **SMC-1**, 364–378 (1971).

[32] Heart Rate Prediction to Monitor Stress Level. https://www.kaggle.com/datasets/vinayakshanawad/heart-rate-prediction-to-monitor-stress-level.

[33] Bisikalo O, Kharchenko V, Kovtun V, Krak I, Pavlov S (2023). Parameterization of the stochastic model for evaluating variable small data in the Shannon Entropy Basis. Entropy.

[34] Tang Y-C, Gottlieb A (2021). Explainable drug sensitivity prediction through cancer pathway enrichment. Sci Rep.

